# Cold Exposure Regulates Hepatic Glycogen and Lipid Metabolism in Newborn Goats

**DOI:** 10.3390/ijms241814330

**Published:** 2023-09-20

**Authors:** Duo Su, Tianhui Zhou, Yan Wang, Linjie Wang

**Affiliations:** 1 Key Laboratory of Livestock and Poultry Multi-Omics, Ministry of Agriculture and Rural Affairs, College of Animal and Technology, Sichuan Agricultural University, Chengdu 611130, China; suduo0821@163.com (D.S.); zth712135@163.com (T.Z.); 2 Farm Animal Genetic Resources Exploration and Innovation Key Laboratory of Sichuan Province, Sichuan Agricultural University, Chengdu 611130, China; wangyan8108@sicau.edu.cn

**Keywords:** cold exposure, glycogen metabolism, goats, lipid metabolism, liver

## Abstract

Cold exposure influences liver metabolism, thereby affecting energy homeostasis. However, the gene regulatory network of the liver after cold exposure remains poorly understood. In this study, we found that 24 h cold exposure (COLD, 6 °C) increased plasma glucose (GLU) levels, while reducing plasma non-esterified fatty acid (NEFA) and triglyceride (TG) levels compared to the room temperature (RT, 25 °C) group. Cold exposure increased hepatic glycogen content and decreased hepatic lipid content in the livers of newborn goats. We conducted RNA-seq analysis on the livers of newborn goats in both the RT and cold exposure groups. A total of 1600 genes were identified as differentially expressed genes (DEGs), of which 555 genes were up-regulated and 1045 genes were down-regulated in the cold exposure group compared with the RT group. Cold exposure increased the expression of genes involved in glycolysis, glycogen synthesis, and fatty acid degradation pathways. These results can provide a reference for hepatic lipid and glycogen metabolism in newborn goats after cold exposure.

## 1. Introduction

Livestock and poultry suffer from cold stress caused by low temperatures, resulting in slow growth, disease, and even death [1]. Cold exposure is a common stressor for newborn livestock. Newborn lambs are particularly susceptible to hypothermia-induced mortality, which accounts for the majority of sheep production losses [2]. A previous study reported that hypothermia induced by cold stress can lead to hypoxia, hypoglycaemia, and metabolic acidosis in lambs [3]. Additionally, the cold environment affects the growth performance, antioxidant status, immune function and expression of related genes in lambs [4]. Based on the various adverse effects of cold stress on newborn goats, this study aims to explore the regulatory effect of cold exposure on hepatic metabolism in newborn goats. During cold stress, aminophylline is shown to increase the metabolic rate in lambs at doses of 16 and 32 mg/kg, and to be a potential therapy for hypothermia or to aid recovery from hypothermia [5]. It has been reported that oxidative damage, disorder, and protein synthesis inhibition can be caused by cold exposure in rats [6]. Cold stress in rats disrupts the balance between oxidative/antioxidant systems in the liver and decreases antioxidant enzyme activity [7]. In addition, cold exposure induces oxidative metabolism and heat production, along with the elevated production of oxidative stress and reactive oxygen species (ROS) in the livers of rats [8]. Cold stress also enhances specific high-density lipoprotein (HDL) activity and alters lipid composition in plasma of mice and humans [9].

The liver is an important organ in mammals, involved in various physiological functions, such as detoxification, protein synthesis, glucose homeostasis, and nutrient utilization [10]. In the liver, the excess glucose in the circulation is used to make fatty acids, while the liver can utilize the stored glycogen or the production of glucose from precursors such as alanine, lactic acid, and glycerol [11]. In mice, cold exposure enhances the mitochondrial tricarboxylic acid cycle and retinol metabolism pathways in the liver but has no significant effect on oxidative phosphorylation [12]. In piglets, the O-GlcNAcylation and apoptosis of the liver are increased after cold exposure. Further results indicate that cold stress regulates liver glucose metabolism and cell apoptosis through the O-GlcNAc/Akt pathway to counter the effects of cold stress [13].

Our previous study has shown that cold exposure increases lipolysis and fatty acid metabolism, and increases the expression of thermogenesis genes in goat brown adipose tissue, suggesting that cold exposure induces glycerolipid and glycerophospholipid metabolism in newborn goats [14]. However, the molecular mechanism underlying the regulation in the liver of newborn goats by cold exposure remains unclear. The aim of the present study was to assess the effects of cold exposure on plasma biochemical indexes and hepatic gene expression profiles, as well as hepatic glycogen and lipid metabolism, in newborn goats. This study provides insight into the understanding of cold exposure on liver metabolism of newborn goats.

## 2. Results

### 2.1. Effects of Cold Exposure on Plasma Biochemical of Newborn Goats

**Figure 1:**
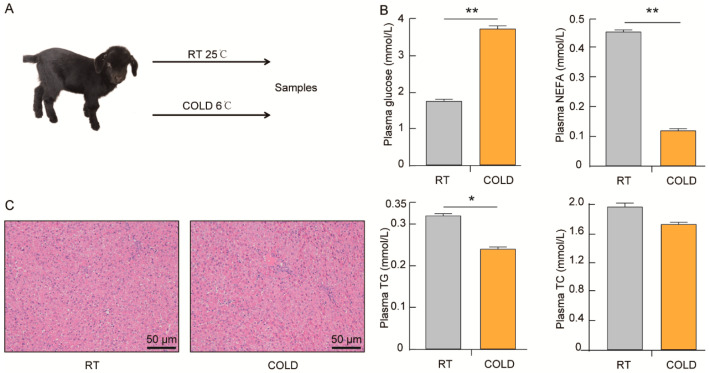
Cold exposure results in increased levels of glucose but decreased levels of NEFA and TG in plasma. (**A**) Experimental design for cold exposure. Livers were isolated from newborn goats maintained at room temperature (RT, 25 °C) or in a cold environment (COLD, 6 °C) for 24 h (n = 4). (**B**) Glucose, non-esterified fatty acids (NEFA), triglycerides (TG), and total cholesterol (TC) levels in plasma (n = 4). (**C**) H&E staining in liver of the RT and COLD groups newborn goats. *p*-values were calculated using Student’s *t*-test; * *p* < 0.05, ** *p* < 0.01.

The aim of this study was to explore the effect of cold exposure on glucose/glycogen metabolism and lipid metabolism in the liver of newborn goats. We maintained newborn goats at either room temperature (RT, 25 °C) or in a cold environment (COLD, 6 °C) for 24 h (Figure 1A). The plasma of goats from the RT and the cold groups was collected for biochemical analysis. The results showed that there were no significant differences in the levels of albumin (ALB), alanine aminotransferase (ALT), and aspartate transaminase (AST) between the two groups. In addition, the concentrations of high-density lipoprotein cholesterol (HDL-C), low-density lipoprotein cholesterol (LDL-C), and total cholesterol (TC) in the plasma of newborn goats were not affected by cold exposure (Figure 1B, Supplementary Table S1). Interestingly, cold exposure increased the level of glucose (GLU) but decreased the level of non-esterified fatty acids (NEFA) and triglycerides (TG) compared with the RT group (Figure 1B, *p* < 0.05). In addition, H&E staining results revealed no significant difference in the morphological analysis of the livers (Figure 1C). The results suggest that cold exposure may impact the glucose and lipid metabolism of newborn goats.

### 2.2. Cold Exposure Changed the Gene Expression Pattern in Liver of Newborn Goats

**Figure 2:**
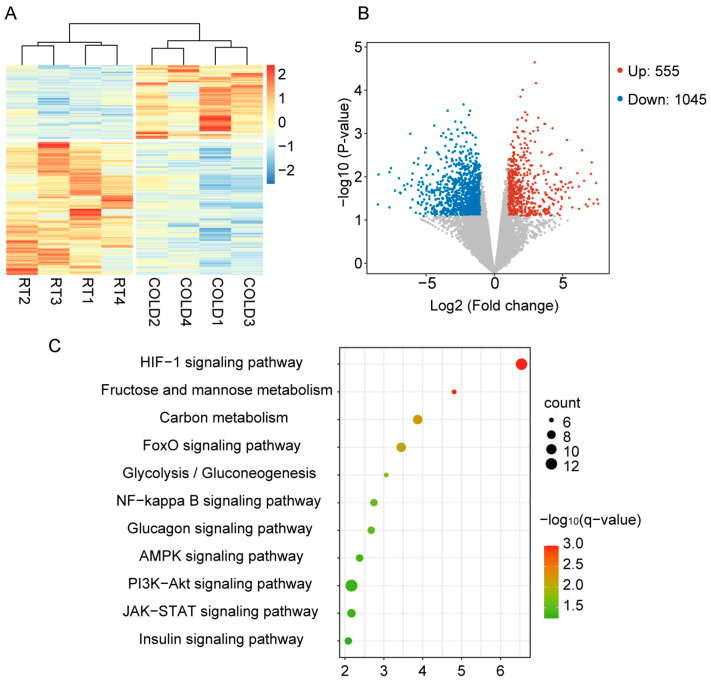
RNA-seq analysis revealed the altered gene expression pattern in livers of newborn goats. (**A**) Heatmap showing hierarchical clustering of gene expression. (**B**) Volcano plot showing DEGs between RT and COLD groups. The red dots represent significantly up-regulated genes, the green dots represent significantly down-regulated genes, and the gray dots represent no difference change genes. (**C**) KEGG pathway analysis showing the enrichment of functional categories (n = 4).

A total of 61.58 gigabases (Gb) of clean data were generated from eight cDNA libraries, and the percentage of Q30 bases was greater than 95.27%. The proportion of reads mapped to the goat reference genome ranged from 96.39% to 96.98%, indicating that the quality of the sequences is sufficient for use as reference data for further analyses. To investigate differences in gene expression profiles between the RT and cold exposure groups, we performed hierarchical clustering analysis on eight samples. Cluster analysis divided the samples for the RT and cold exposure groups into two major clusters (Figure 2A), indicating the overall expression profile of genes of the liver tissues was significantly altered after cold exposure. To further clarify the details of the gene expression profiles between the RT and cold exposure groups, the DESeq2 R package was used to identify differentially expressed genes (DEGs). A total of 1600 DEGs were identified, of which 555 genes were up-regulated, and 1045 genes were down-regulated in the cold group compared with the RT group (Figure 2B, Supplementary Table S2). KEGG pathway analysis of the up-regulated genes by cold exposure revealed significant enrichment in several major metabolic pathways, including the HIF-1 signaling pathway, glycolysis/gluconeogenesis, glucagon signaling pathway, carbon metabolism, fructose and mannose metabolism, FoxO signaling pathway, and AMPK signaling pathway (Figure 2C). These results indicate that cold exposure caused dramatic changes in the gene expression profiles.

### 2.3. Cold Exposure Regulates Glycogen Metabolism in Liver of Newborn Goats

**Figure 3:**
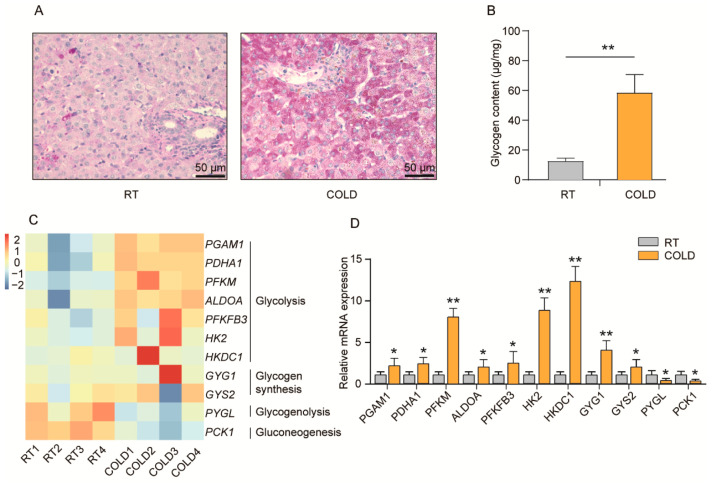
Cold exposure increased hepatic glycogen deposition in newborn goats. (**A**) PSA staining in livers of the RT and COLD groups newborn goats. (**B**) The glycogen content in livers of the RT and COLD groups of newborn goats (n = 4). (**C**) Heatmap showing the differentially expressed genes related to glycolysis, glycogen synthesis, glycogenolysis and gluconeogenesis pathways (n = 4). (**D**) qPCR analysis of *PGAM1*, *PDHA1*, *PFKM*, *ALDOA*, *PFKFB3*, *HK2*, *HKDC1*, *GYG1*, *GYS2*, *PYGL* and *PCK1* in livers after cold exposure (n = 4). *p*-values were calculated using Student’s *t*-test; * *p* < 0.05, ** *p* < 0.01.

The aim of this study was to investigate the effects of cold exposure on glucose metabolism in the livers of newborn goats. The PSA staining results showed that there was a higher hepatic glycogen content after cold exposure (Figure 3A). Further detection results found that the glycogen content was significantly increased in the livers of newborn goats after cold exposure compared to the RT group (Figure 3B, *p* < 0.01). These findings suggest that cold exposure promotes the deposition of glycogen in the livers of newborn goats, indicating a potential influence on glucose metabolism. Furthermore, RNA-seq results revealed that cold exposure significantly activated the glycolysis and glycogen synthesis pathways in the liver, such as *PGAM1*, *PDHA1*, *PFKM*, *ALDOA*, *PFKFB3*, *HK2*, *HKDC1*, *GYG1*, and *GYS2*. Notably, *HKDC1* and *PFKM* are two key rate-limiting enzymes in the glycolytic pathway, while *GYS2* is a major enzyme for glycogen synthesis in the liver (Figure 3C, *p* < 0.05). In contrast, cold exposure significantly decreased the expression of the glycogenolysis pathway-related gene *PYGL* (*PYGL* is the main rate-limiting enzyme in glycogenolysis) and the gluconeogenesis pathway-related gene *PCK1* was also down-regulated under cold exposure (Figure 3C, *p* < 0.05). The qPCR results provide robust evidence that cold exposure significantly up-regulated the expression of glycolysis and glycogen synthesis pathway-related genes, while down-regulated the expression of glycogenolysis and gluconeogenesis pathway-related genes (Figure 3D, *p* < 0.05). These results suggest that cold exposure induces glucose metabolism by promoting glycogen synthesis and glycolysis, inhibiting glycogenolysis and gluconeogenesis, and thus increasing hepatic glycogen deposition (figure in below).

### 2.4. Cold Exposure Regulates Lipid Metabolism in Liver of Newborn Goats

**Figure 4:**
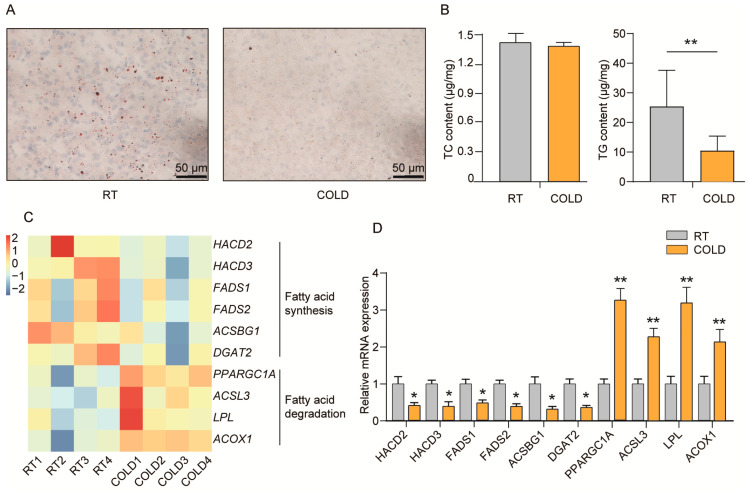
Cold exposure reduces hepatic lipid deposition in newborn goats. (**A**) Oil red O staining in livers of the RT and COLD groups newborn goats. (**B**) The TC and TG content in livers of the RT and COLD groups newborn goats (n = 4). (**C**) Heatmap showing the differentially expressed genes related to fatty acid synthesis and fatty acid degradation pathways (n = 4). (**D**) qPCR analysis of *HACD2*, *HACD3*, *FADS1*, *FADS2*, *ACSBG1*, *DGAT2*, *PPARGC1A*, *ACSL3*, *LPL* and *ACOX1* in livers after cold exposure (n = 4). *p*-values were calculated using Student’s *t*-test; * *p* < 0.05, ** *p* < 0.01.

This study also aimed to determine whether cold exposure affects hepatic lipid metabolism of newborn goats. Oil red O staining showed that lipid droplets in the livers of newborn goats were significantly reduced after cold exposure (Figure 4A). Next, we measured the TC and TG content in the livers of newborn goats. We found that TC content was not affected after cold exposure while the TG level was significantly decreased after cold treatment (Figure 4B, *p* < 0.01), suggesting that cold exposure reduced lipid deposition in the livers of newborn goats compared with the RT group.

**Figure 5:**
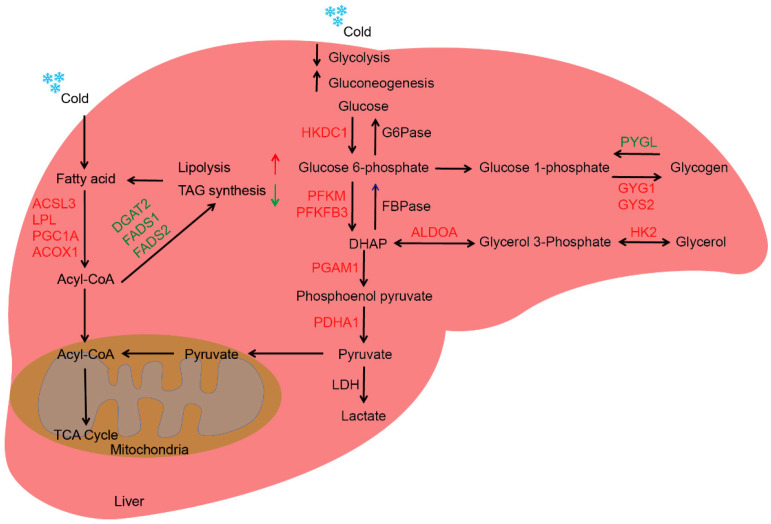
A working model showing that cold exposure regulates lipid and glycogen metabolism in livers of newborn goats. Cold exposure induces glucose metabolism by promoting glycogen synthesis and glycolysis, inhibiting glycogenolysis and gluconeogenesis, and thus increasing hepatic glycogen deposition. In addition, Cold exposure also reduces hepatic lipid deposition by promoting fatty acid degradation and inhibiting fatty acid synthesis. Red represents up-regulated genes. Green represents down-regulated genes. Blue snowflakes indicated cold exposure of newborn goats for 24 h.

RNA-seq analysis revealed that several pathways related to lipid metabolism were significantly altered by cold exposure. Specifically, the expression of genes involved in fatty acid elongation, such as *HACD2* and *HACD3*, was significantly down-regulated after cold exposure (Figure 4C, *p* < 0.05). Additionally, genes related to fatty acid synthesis, including *FADS1*, *FADS2*, and *ACSBG1*, were also suppressed. *FADS1* and *FADS2* are two key enzymes in de novo fatty acid synthesis. *DGAT2*, which inhibits the final step of TG synthesis, was significantly down-regulated after cold exposure. On the other hand, genes involved in fatty acid degradation, such as *PPARGC1A*, *ACSL3*, *LPL*, and *ACOX1*, were significantly up-regulated after cold exposure (Figure 4C, *p* < 0.05). *ACOX1* is the first enzyme in the fatty acid oxidation pathway, while *LPL* is a key gene in the process of TG degradation to fatty acids. The *ACSL3* gene encodes a long-chain acyl α synthetase, which is a key enzyme in β-oxidation. The qPCR results provide robust evidence that cold exposure significantly down-regulated the expression of fatty acid synthesis pathway-related genes, while up-regulated the expression of fatty acid degradation pathway-related genes (Figure 4D, *p* < 0.05). These findings suggest that cold exposure reduces hepatic lipid deposition in newborn goats by promoting fatty acid degradation and inhibiting fatty acid synthesis (Figure 5).

## 3. Discussion

In mammals, long-term exposure to cold can cause a variety of physiological reactions, such as severe energy depletion, lack of energy substrates, and increased glucose production in the liver [15]. However, the liver, as the main organ, participates in adaptation regulation, maintains glucose homeostasis, and plays a key role in energy metabolism. In this study, we found that 24 h cold exposure increased the level of plasma glucose but decreased the level of plasma NEFA and TG compared with the RT group. Cold exposure also significantly increased glycogen content and significantly decreased lipid deposition in the livers of newborn goats. The RNA-seq results showed that cold exposure increases hepatic glycogen deposition by promoting glycogen synthesis and glycolysis, while reducing hepatic lipid deposition by promoting fatty acid degradation. Thus, we hypothesized that newborn goats mobilized fat reserves to store glucose and meet the increased heat production needs at low temperatures.

After three weeks of low temperature exposure, the plasma glucose level was increased and the plasma triglyceride level was decreased in dairy goats [16], consistent with the results after cold exposure 24 h in newborn goats. There was a slight, but not significant increase in hepatic glucose output after feeding, and there was no effect on blood glucose concentration [17]. Exposure to cold environments increased the output of glucose in the liver, which may explain the higher blood glucose levels in cold-treated goats. In mice, cold exposure reduced plasma TG concentrations but had no effect on plasma cholesterol concentrations [18], consistent with our results. It was found that cold exposure did not affect aspartate transaminase (AST), alanine transaminase (ALT), alkaline phosphatase (ALP) or total bilirubin (TB) levels in the plasma of Yorkshire pigs [19]. Similarly, 15-day-old cocks were exposed to 12/−1 °C acute (24 h) cold stress and chronic (20 d) cold stress, respectively. The contents of insulin and NEFA in the plasma of the cocks subjected to acute cold stress showed fluctuation, while the glucose contents increased first and then decreased. The contents of NEFA and glucose in the plasma of the cocks subjected to chronic cold stress increased gradually with a time course trend [20]. Our results showed that cold exposure increased the level of glucose and decreased the NEFA in plasma compared with the RT group. However, cold exposure does not affect the levels of TG, TC, HDL-C and LDL-C ALT, and AST in plasma.

Previous studies have shown that mice exposed to cold (4 °C) for up to 5 days significantly reduced the levels of TG and TC in the liver and increased the expression of gluconeogenic genes [21]. At the same time, acute cold exposure increased the consumption of liver glycogen and increased protein kinase B (AKT) phosphorylation to maintain the hepatocyte energy balance in mice [22]. The glycogen content of weaned piglets at 21 days of age was significantly increased under acute cold exposure [23]. In the present study, we found that the liver glycogen content of newborn goats was increased significantly (*p* < 0.01) and the TG content decreased significantly (*p* < 0.01) after 24 h cold exposure, which was consistent with the results of mice and weaned piglets, indicating that cold exposure can increase liver glycogen content and decrease TG content.

Furthermore, *PFKFB3*, *PDHA1*, *HK2*, *ALDOA*, *PGAM1*, *HKDC1*, and *PFKM* genes, which are involved in the glycolysis pathway, were up-regulated under cold stress. Hexokinase (HK) catalyzes the phosphorylation of glucose, the first rate-limiting enzyme or key enzyme of the glycolysis pathway [24]. *HKDC1* is one of the isoforms expressed by *HK* in the liver, which has low glucose phosphorylation ability and has proved its association with hepatocyte mitochondria. *HKDC1* gene deletion leads to changes in liver TG levels [25]. Phosphofructokinase (PFKM) is the second rate-rater of the glycolysis pathway and a protein-coding gene that catalyzes the phosphorylation of fructose-6-phosphate to fructose-1, 6-diphosphate [26]. *PFKFB3* encodes 6-phosphofructose-2 kinase/fructose-2 and 6-bisphosphatase-3 enzymes (PFK-2/FBASE-2). PFK-2/FBASE-2 is a bifunctional enzyme that controls glycolysis flux through fructose 2, 6-diphosphate (F-2, 6-P). F-2, 6-P is a potent allosteric activator of 6-phosphofructokinase-1 (PFK-1) that triggers aerobic oxidation of glucose metabolism. Recent studies have reported that PFKFB3 regulates inflammation induced by a high-fat diet (HFD) and inflammation associated with overnutrition [27].

However, our results show that cold exposure promoted the glycolysis of newborn goats to meet the high energy needs of newborn goats. Phosphorylase kinase (PHKA2) degrades glycogen to produce glucose 1-phosphate, which is a key enzyme in glycogen decomposition. A lack of the *PHKA2* gene has been reported to cause glycogen storage disease [28]. Glycogen synthase 2 (GYS2) is considered a key enzyme involved in the regulation of glycogen synthesis. It is part of the rate-limiting step in catalyzing glycogen synthesis and transfer of glucose molecules from uridine diphosphate (UDP)-glucose to the terminal branch of glycogen molecules. Glycogen 1 (GYG1) is a glycosyltransferase that catalyzes the formation of short glucose polymers in the auto-glycation of glucose uridine diphosphate. The polymer extends to form glycogen under the catalysis of glycogen synthetase (GYS2) and branched enzymes [29]. In this study, cold exposure induced the expression of *GYG1*, which promotes glycogen synthesis in the liver by regulating glycolysis metabolism, which is consistent with our phenotypic results. This study found that cold exposure can affect the glucose metabolism of newborn goats. Our results demonstrate that the glycolysis/gluconeogenesis pathway was activated after cold in the livers of newborn goats.

The liver is an essential organ for the metabolism of lipids. Fatty acid synthesis is a complex process due to the different lengths of fatty acid chains. Lipid metabolism is a key biological process of lipid synthesis and degradation in animals. Diacyl glyceryl transferase-2 (DGAT2) catalyzes the final reaction of TG synthesis using diacylglycerol and fatty acyl-CoA as substrates [30]. HACD3 catalyzes the dehydration of 3-hydroxy acyl-coA intermediates to trans—2, 3-dilute acyl-CoA, catalyzes the extended circulation of long-chain fatty acids, and promotes the transport and synthesis of unsaturated fatty acids (UFAs) [31]. Fatty acid desaturase 1 (FADS1), as a key enzyme in the metabolism of polyunsaturated fatty acids (PUFA), catalyzes di-high -y-linolenic acid (DGLA) into arachidonic acid (AA) [32]. The results showed that the cold group had reduced liver fat content compared to the RT group. The RNA-seq results showed that cold exposure significantly down-regulated the genes related to liver fatty acid synthesis (*DGAT2*, *HACD3*, *FADS1*, *ACSBG1*). It showed that fatty acid and TG synthesis were inhibited in the liver of newborn goats after cold exposure. Previous studies have shown that ACSLs catalyze the conversion of free long-chain fatty acids to fatty acid acyl-CoA, which plays a key role in lipid synthesis and fatty acid degradation [33]. The expression of *ACSL3* increased in the liver of hamsters fed a diet rich in fat and cholesterol [34]. According to the RNA-seq results, we found that *ACSL3* expression was also increased in the cold group compared with the RT group. LPL is a key gene in the process of degrading TG into fatty acids [35]. Previous studies have shown significant increases in *LPL* expression in NASH livers in both humans and mice [36]. Our results indicate that the fatty acid β oxidation pathway is activated and the fatty acid synthesis pathway is down-regulated during cold exposure. Finally, we recognize the limitations of our research. Although we investigated the effects of cold exposure on hepatic glycogen and lipid metabolism in newborn goats, the effects of cold exposure on hepatic metabolism in adult goats still need to be explored in future research.

## 4. Materials and Methods

### 4.1. Ethics Statement

All research involving animals was conducted according to the regulation proposed by the Institutional Animal Care and Use Committee at Sichuan Agricultural University, under permit No. DKY-2022102011.

### 4.2. Animals and Sample Collection

All animals were raised at the breeding center of Sichuan Agricultural University, Ya’an, China. Female Chuanzhong black goats (n = 16) were artificially inseminated with the semen of a ram. Then, there were 17 pregnant ewes lambing, including 9 males and 11 females. After birth, the newborn goats were wiped and fed colostrum (30 mL/kg body weight) in a 25 °C environment for 2 h. A total of 8 male kids were selected and randomized into room temperature (n = 4) and cold exposure groups (n = 4). At 2 h of age, kids from the 25 °C environments were placed in a 6 °C cold room (COLD, 6 °C) or maintained at room temperature (RT, 25 °C) for 24 h. Warmed colostrum was fed three times at 8, 14, and 20 h of age. After 24 h, blood samples from newborn goats were collected from the RT and cold exposure groups, respectively, and the samples were then centrifuged at 1000× *g*, 4 °C for 15 min and stored at −20 °C for plasma biochemical analysis. Finally, the newborn goats from the RT and cold exposure groups were anesthetized by intraperitoneal injection of pentobarbital sodium (60 mg/kg) and ketamine (80 mg/kg), and the liver tissues were collected and stored at −80 °C.

### 4.3. Plasma Biochemical Analysis

The contents of glucose (GLU), triglycerides (TG), total cholesterol (TC), low-density lipoprotein cholesterol (LDL-C), high-density lipoprotein cholesterol (HDL-C), non-esterified fatty acids (NEFA), aspartate transaminase (AST), alanine transaminase (ALT), and albumin (ALB) in the plasma of newborn goats were determined using Hitachi 7020 Automated Biochem (Hitachi, Tokyo, Japan).

### 4.4. Hematoxylin-Eosin (H&E), Periodic Acid Schiff (PAS), and Oil Red O Staining

For H&E staining, the liver tissues were fixed in 4% formaldehyde, embedded with paraffin, and cut into sections (4-µm thick). Next, liver sections were stained with a hematoxylin–eosin (H&E) staining kit (G1120, Solarbio, Beijing, China) according to the manufacturer’s instructions. Finally, liver sections were observed and photographed under a light microscope (Olympus, Tokyo, Japan). For glycogen staining, liver tissues were fixed in 4% paraformaldehyde, embedded in paraffin, and cut into 4 μm sections. Then, they were stained with periodic acid–Schiff (PAS). For oil red O staining, liver tissues were embedded using an OCT embedding agent. Then, dye sections were stained with oil red working solution for 10 min. The nuclei were counterstained with hematoxylin for 3–5 min, then covered with filter paper to remove the surrounding water, and sealed with glycerin gelatin. The sections were imaged under an inverted microscope (Olympus, Tokyo, Japan).

### 4.5. Glycogen, Triglyceride (TG), and Total Cholesterol (TC) Analysis

The glycogen contents of the livers were measured using the Liver Glycogen Assay Kit (A043-1-1, Jiancheng, Nanjing, China) according to the manufacturer’s instructions. Briefly, we mixed 50 mg of the sample with a hydrolysis buffer, and incubated it at 100 °C for 20 min. The color substrate solution was then added. The absorbance of the prepared samples was measured at 620 nm using the Varioskan LUX Microplate Reader (Thermo Fisher Scientific, Waltham, MA, USA).

The liver tissues were weighed according to weight (g):volume (mL) = 1:9 and added to 9 times the volume of anhydrous ethanol. The samples were homogenized and centrifuged at 2500 rpm for 10 min. The supernatant was collected for the assay. TG and TC were determined by GPO-PAP and COD-PAP with the Triglyceride Assay Kit and Total Cholesterol Assay Kit (A110-1 and A111-1-1 Jiancheng, Nanjing, China) according to the manufacturer’s instructions. The absorbance of the prepared samples was measured at 510 nm and 500 nm, respectively, using the Varioskan LUX Microplate Reader (Thermo Fisher Scientific, Waltham, MA, USA).

### 4.6. Quantitative Real-Time PCR

Total RNA was extracted with TRIzol reagent (Invitrogen Life technologies, Carlsbad, CA, USA) from liver tissues according to the manufacturer’s protocols. The RNA was reverse-transcribed into cDNA using the HiScript III RT SuperMix (Vazyme, Nanjing, China). The stable expression housekeeping gene *GAPDH* in the RT and cold-treated groups using transcriptome data was used as an internal reference to calculate the relative gene expression. Bio-rad CFX 96 quantitative PCR was used to analyze the relative expression levels of each gene by the 2^−ΔΔCT^ method. The qPCR primer sequences are summarized in Supplementary Table S3.

### 4.7. RNA Library Construction, and Sequencing

All samples revealed an RNA integrity number (RIN) above 8.5. Sequencing libraries were generated using the NEBNext UltraTM RNA Library Prep Kit for Illumina (NEB, USA). Magnetic beads with Oligo (dT) were used to enrich mRNA with a polyA structure. DNA libraries were sequenced on an Illumina Novaseq 6000 platform and 150 bp paired-end reads were generated.

The RNA-seq data reported in this paper have been deposited in the Genome Sequence Archive (Genomics, Proteomics & Bioinformatics 2017) in National Genomics Data Center (Nucleic Acids Res 2020), Beijing Institute of Genomics, Chinese Academy of Sciences, under the accession number CRA010578 and are publicly accessible at https://bigd##big##ac##cn/gsa (accessed on 10 April 2023).

### 4.8. RNA-Seq Analysis

The RNA-seq clean reads were aligned to the goat reference genome (ARS1) using HISAT2 (v2.2.1), and the reads were quantified using featureCounts within the Rsubread package (v2.8.1). DEGs were identified as genes with a|log2fold change| ≥ 1 and *p* < 0.05. KEGG functional enrichment analysis of the DEGs was performed using Metascape (http://metascape.org/ (accessed on 25 July 2023)). Terms with *p* < 0.01 were considered significantly enriched for DEGs.

### 4.9. Statistical Analysis

Statistical analyses were conducted in SPSS Statistics 19.0. All data are presented as the mean ± standard deviation (SD) of the replicates from independent experiments unless stated otherwise. *p*-values were calculated using Student’s *t*-test. *p* < 0.05 was considered statistically significant, and *p* < 0.01 was considered highly statistically significant (* *p* < 0.05; ** *p* < 0.01).

## 5. Conclusions

In this study, we found that cold exposure increased the level of plasma glucose but decreased the level of plasma NEFA and TG compared with the RT group. Cold exposure increased hepatic glycogen content and decreased hepatic lipid content in the livers of newborn goats. Cold exposure increased the expression of genes involved in glycolysis, glycogen synthesis, and fatty acid β-oxidation pathways. These results can provide a reference for hepatic lipid and glycogen metabolism in newborn goats after cold exposure.

## Data Availability

The datasets presented in this study can be found in online repositories. The names of the repository/repositories and accession number(s) can be found below: Genome Sequence Archive under accession number CRA010578.

## Supplementary Materials

The following supporting information can be downloaded at: https://www.mdpi.com/article/10.3390/ijms241814330/s1.
